# Woody Plant Encroachment has a Larger Impact than Climate Change on Dryland Water Budgets

**DOI:** 10.1038/s41598-020-65094-x

**Published:** 2020-05-15

**Authors:** Adam P. Schreiner-McGraw, Enrique R. Vivoni, Hoori Ajami, Osvaldo E. Sala, Heather L. Throop, Debra P. C. Peters

**Affiliations:** 1 School of Earth and Space Exploration, Arizona State University, Tempe, AZ 85287 USA; 2 Department of Environmental Sciences, University of California, Riverside, CA 92587 USA; 3 School of Sustainable Engineering and the Built Environment, Arizona State University, Tempe, AZ 85287 USA; 4 School of Life Sciences, Arizona State University, Tempe, AZ 85287 USA; 5 Global Drylands Center, Arizona State University, Tempe, AZ 85287 USA; 6 School of Sustainability, Arizona State University, Tempe, AZ 85287 USA; 7 USDA-ARS, Las Cruces, New Mexico 88001 USA

**Keywords:** Ecosystem services, Hydrology

## Abstract

Woody plant encroachment (WPE) into grasslands is a global phenomenon that is associated with land degradation via xerification, which replaces grasses with shrubs and bare soil patches. It remains uncertain how the global processes of WPE and climate change may combine to impact water availability for ecosystems. Using a process-based model constrained by watershed observations, our results suggest that both xerification and climate change augment groundwater recharge by increasing channel transmission losses at the expense of plant available water. Conversion from grasslands to shrublands without creating additional bare soil, however, reduces transmission losses. Model simulations considering both WPE and climate change are used to assess their relative roles in a late 21^st^ century condition. Results indicate that changes in focused channel recharge are determined primarily by the WPE pathway. As a result, WPE should be given consideration when assessing the vulnerability of groundwater aquifers to climate change.

## Introduction

Global drylands covering nearly 40% of the Earth’s land surface have been dramatically transformed by woody plant encroachment (WPE)^1–4^. Managed grazing is the predominant use of drylands, making it the single most extensive form of land use on the planet^1^. As such, WPE into grasslands is often considered a negative outcome since it may reduce forage production for livestock^5^ and decrease habitat for native species^6^. As shrubs become dominant in a dryland ecosystem, processes in the water and energy budgets are affected^7–9^, including groundwater recharge^10^. Most prior observational^11,12^ and modeling^13^ efforts indicate that WPE reduces diffuse vertical recharge due to water uptake by deeply rooted woody plants^12,14^. However, these cases have focused on flat areas that lack topographic effects on water transport. Where terrain controls are important^15^, WPE could potentially have local and downstream consequences on both vertical and lateral water exchanges. Nevertheless, little evidence is available on downstream consequences of WPE within interconnected hillslope and channel systems^16^, even though many arid landscapes consist of these topographic features.

In addition to WPE, directional changes in climate are expected to impact water budgets in drylands^17^. Prior studies in these regions predict drying caused by increased temperatures and higher evapotranspiration (*ET*)^18–20^, in some cases with decreases in precipitation^21,22^. In addition to directional changes in precipitation and temperature, changes to precipitation frequency, intensity, and seasonal distributions are expected^23^. Recent work has shown that increases in precipitation variability are more important than changes to the mean annual precipitation in impacting groundwater recharge in dryland playa lakes^24^. Prior observations and model simulations also suggest significant climate change impacts of extreme precipitation events^25,26^. These impacts are critical because extreme storm events with a high magnitude or intensity can generate streamflow and channel transmission losses. For instance, channel losses contribute up to 40% of recharge to arid and semiarid aquifers^27^, and these losses are commonly used as proxies for recharge^28^. While conceptual models of the hydrologic impact of WPE and climate change have been proposed^7^, studies on their combined effects on channel transmission losses are lacking. Indeed, it is unknown if WPE and climate change will interact in a linearly additive manner or in a nonlinear way to affect groundwater recharge in drylands.

**Figure 1:**
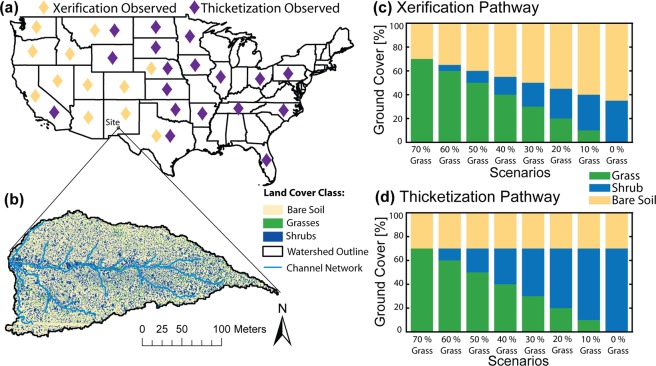
Model scenarios in the context of continental scale WPE. (**a**) Study site location along with WPE pathways in the United States. Diamonds indicate states where woody plant encroachment, via xerification (yellow) or thicketization (purple), has been observed^27^. (**b**) Current spatial pattern of grasses, shrubs, and bare soils (2013) at the watershed study site. Stacked bar plots illustrate WPE model scenarios for the xerification pathway (**c**) and the thicketization pathway (**d**).

In the U.S., WPE occurs via two pathways (Fig. 1a): ‘xerification’ is where grass loss associated with WPE leads to an increase in bare soil cover, while ‘thicketization’ involves the replacement of grasses by shrubs without a simultaneous increase in bare soil^29^. In dry environments where water resources are limited, grassland plants tend to be smaller and more tightly spaced together than do shrublands. Gaps between shrubs allow resource removal by wind^30^ and water^31^ transport, which increases the bare soil coverage. Mean annual precipitation is a good predictor for the occurrence of each pathway, with drier areas (<400 mm per year) in the western U.S. undergoing xerification^32,33^, while wetter sites in the central and eastern U.S. have experienced primarily thicketization^34,35^. This distinction is important as these pathways directly affect the connectivity of surface flow over bare soil patches, and the lateral subsurface water movement on hillslopes^36^, which in turn impact focused recharge in downstream channels^37,38^. Since channel losses depend on hillslope processes affected by plants, a modeling approach that can account for both terrain and vegetation patch effects on hydrologic connectivity is needed. Similar pathways of WPE can be found globally^39^ and have been identified in Africa^40^, South America^41^, and China^42^, although the precipitation thresholds indicating which pathway is more likely are not clear.

We applied a distributed, process-based ecohydrological model to determine the hydrologic impacts of WPE and climate change. The investigation was performed using data from a small, densely instrumented watershed (4.6 ha) in the Chihuahuan Desert (Fig. 1b) where historical WPE has been well documented^43–45^. We conducted a series of simulation scenarios using observed meteorological data over a 6.25-year period (using all available data) to quantify impacts of xerification (Fig. 1c) and thicketization (Fig. 1d) pathways by varying the percentages of shrub, grass, and bare soil cover. We then used a stochastic downscaling approach to construct synthetic time series of meteorological forcings based on historical (1990-2005) and late century (2085-2100) conditions projected from three general circulation models (GCMs) and one greenhouse gas emissions scenario (RCP 8.5). Our analysis focuses on the consequences of the combined effects of WPE and climate change on the evapotranspiration and channel transmission losses that form the major parts of the dryland water budget.

With this framework, we address the following questions about the conversion of grasslands to shrublands: (1) What are the impacts of woody plant encroachment on the dryland water budget? (2) Do varying WPE pathways lead to different water budget components?, and (3) What are the relative roles of WPE and projected climate conditions on channel transmission losses at the end of the 21^st^ century?

### Diverging effects of WPE on water budget components

**Figure 2:**
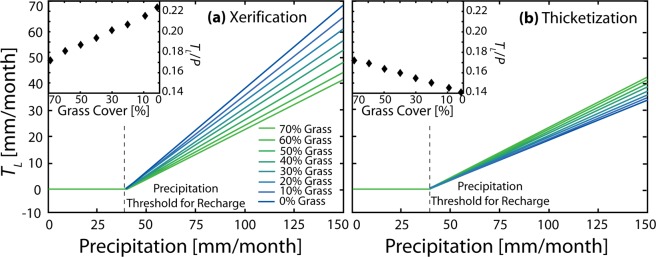
Impacts of two woody plant encroachment pathways on transmission losses. Relation between monthly transmission losses (*T*_*L*_) and precipitation (*P*) for (**a**) xerification and (**b**) thicketization pathways. Insets display annual *T*_*L*_/*P* relation with percent grass cover (%).

Simulations using the 6.25-year observed forcings (Fig. S1) with a calibrated model (see Methods) show that WPE via the xerification pathway increased focused recharge in channel features during summer months with high precipitation (Fig. 2a). While WPE does not affect the precipitation threshold necessary for recharge (40 mm/month in this system) among various xerification scenarios, the annual ratio of transmission losses to precipitation (*T*_*L*_/*P*) increases with lower grass cover, equivalent to +13 mm per year or +29% for the lowest grass cover scenario relative to an initial grassland. Due to the higher bare soil cover during xerification, larger amounts of infiltration-excess overland flow from hillslopes reach the channel network^46^, augmenting channel transmission losses that lead to focused recharge^37^. This result is consistent with prior studies showing a reduction of infiltration with increases in bare soil^47^ at both the plot and field scales^48^ as well as increases in the hydrologic connectivity of hillslopes and channel systems during xerification^49,50^.

While increases in focused recharge caused by xerification are linear, other water budget components exhibit nonlinearities with a reduction in grass cover (Table 1). Reductions in *ET* are due to the lower soil infiltration caused by higher bare soil, yielding large declines of annual *ET/P* from 81 to 74%. In dryland settings, *ET* is water limited, such that this trend reflects a decrease in plant available water with increasing *T*_*L*_. Indeed, the high aridity in the study watershed results in all of the *P* that infiltrates into the soil being used for *ET*, irrespective of the type of plant. As a result, changes to watershed *ET* reflect changes to the physical structure of the watershed that impact infiltration of *P* into the soil. It is noteworthy that reductions in *ET* asymptote towards 200 mm per year in the xerification pathway for the lowest grass cover. This asymptote is caused by the increase in *ET* from shrub patches as the total vegetation cover decreases (Fig. S2). Since bare soil patches do not transpire soil water, their expansion with xerification provides more opportunity for overland flow generation in bare soil patches and lateral soil water redistribution to shrub locations.

**Table 1:** Annual water budget components for xerification and thicketization pathways using observed meteorological forcing. Water budget variables: *P* is the precipitation, *ET* is evapotranspiration, *Q* is streamflow at the watershed outlet, and *T*_*L*_ is transmission losses. The mean soil moisture (Mean ϴ) for the 6.25-year period is also presented as interannual changes in soil water storage are negligible.

Scenario	Grass Cover	*P* [mm/yr]	*ET* [mm/yr]	*Q* [mm/yr]	*T*_*L*_ [mm/yr]	Mean ϴ [mm]	*T*_*L*_/*P* [−]
Xerification Pathway	70%	271	220	3	47	19.1	0.173
	60%	271	215	6	49	18.9	0.181
	50%	271	211	8	51	18.8	0.188
	40%	271	207	10	53	18.7	0.195
	30%	271	205	11	55	18.5	0.202
	20%	271	203	11	57	18.4	0.209
	10%	271	201	10	59	18.2	0.216
	0%	271	201	9	61	18.0	0.223
Thicketization Pathway	70%	271	220	3	47	19.1	0.173
	60%	271	221	3	46	19.0	0.169
	50%	271	223	3	44	19.0	0.164
	40%	271	224	3	43	18.9	0.159
	30%	271	225	3	42	18.9	0.154
	20%	271	227	3	40	18.7	0.149
	10%	271	228	3	40	18.7	0.146
	0%	271	230	2	38	18.6	0.142

By increasing infiltration-excess runoff, WPE via the xerification pathway also results in a nonlinear increase in streamflow, *Q* (Table 1). Though annual *Q/P* are typically low (1 to 4%), a notable maximum in streamflow is observed for grass cover between 20 and 30% for the xerification pathway. Since the partitioning between *Q* and *T*_*L*_ is controlled by the interaction between hillslope runoff and the initial channel infiltration capacity^46,51^, this nonlinearity is explained by the variation of hydrograph properties with changes in grass cover. Low grass cover increases the hillslope flow connectivity which results in more overland flow reaching the dry ephemeral channel during a transient period when capillary forces increase infiltration rates (see Methods). This results in higher *T*_*L*_ and lower streamflow for the lowest grass cover cases.

Since WPE does not result in an increase in bare soil along the thicketization pathway, an opposite response to the xerification case is noted. Indeed, *T*_*L*_/*P* shows a linear decrease as grass cover decreases (Fig. 2b, Table 1). This reduction is caused by two mechanisms that increase *ET/P* from 81 to 85%: increased shrub canopy interception of precipitation and increased soil infiltration underneath shrubs as compared to grasses^52,53^. Canopy interception capacity in shrub areas increases by 64% up to a threshold of 0.65 mm due to the higher leaf area index of shrubs^54^, while soil infiltration increases by 66% due to the higher surface hydraulic conductivity underneath shrubs as compared to grasses (Table S1). WPE along the thicketization pathway also reduces streamflow from *Q*/*P* of 1% to 0.8%, though the sensitivity is quite low. While these effects also occur for the xerification pathway, the impact of expanding bare soil cover on runoff production overwhelms other shrub-induced mechanisms, leading to the diverging impacts of the two different encroachment pathways on evapotranspiration and channel transmissions losses.

### Non-stationarity of hydrologic impact of WPE with climate change

**Figure 3:**
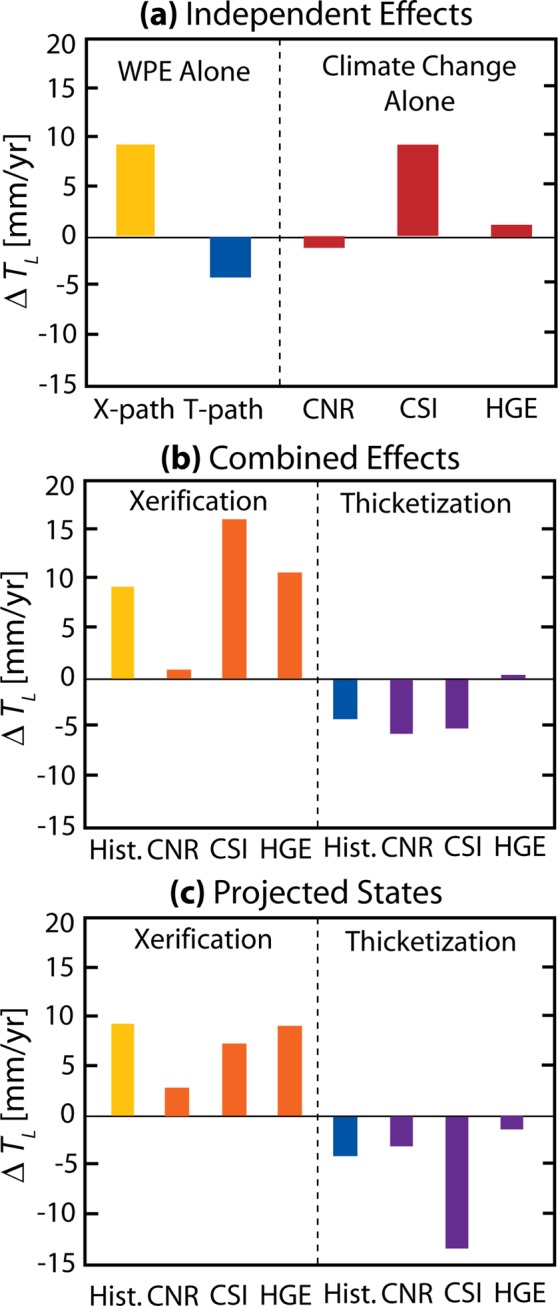
Combined impacts of climate change and WPE on transmission losses. (**a**) The difference in the average annual *T*_*L*_ between a historical grassland and end-member shrubland states forced with historical climate (left) and grassland forced with climate change forcings (right). The X-path is the xerification pathway and the T-path is the thicketization pathway. (**b**) The difference in the average annual *T*_*L*_ between a historical grassland and two shrubland states forced with a historical climate based on NLDAS-2 data (‘Hist.’) or one of 3 climate change projections, CNRM-CM5 (‘CNR’), CSIRO Mk.3.6.0 (‘CSI’), or HadGEM2-ES (‘HGE’). (**c**) The difference in the average annual *T*_*L*_ between shrubland and grassland when both vegetation states use the same meteorological forcings for historical conditions (‘Hist.’) or one of the 3 climate change projections.

We simulated vegetation cover conditions for the final states of the WPE pathways (Fig. 1c,d) and an initial grassland state (70% grass, 30% bare soil) for late 21^st^-century climate conditions (2085-2100) and compared these to a historical climate period parameterized with NLDAS-2 (Fig. S3, Methods). Overall, annual changes in channel transmission losses show that WPE has a stronger impact on dryland water budgets than the worst-case climate change conditions (late-century, RCP 8.5) explored here. As described previously, annual differences in *T*_*L*_ between a historic grassland and final shrub states show diverging outcomes under the two pathways (Fig. 3a). These effects are larger than the climate change impact on a grassland for the three GCMs. Indeed, the WPE cases show a greater sensitivity than the possible future impacts of climate change alone. When the effects are combined (Fig. 3b), the annual change in *T*_*L*_ is not a linearly additive process and the directionality of the future changes are determined almost entirely by the WPE pathway, rather than the magnitude of the imposed climate change signal.

To further explore this outcome, Fig. 3c shows how the WPE pathways will affect *T*_*L*_ for the projected climate conditions at late-century. While the general patterns discussed previously still hold, the sensitivity to WPE varies considerably as compared to the historical period (Fig. 3b). This demonstrates that there is an embedded non-stationarity imposed by climate change on the hydrologic response to WPE for both pathways. Specific changes to *T*_*L*_ for each shrubland state depend on the climate-induced changes in precipitation amount and patterns^55^. For instance, the CSIRO projected climate (reduced total precipitation, increased precipitation intensity) shows large impacts to the annual *T*_*L*_ changes, with a reduced sensitivity for the xerification, and increased sensitivity for the thicketization pathways, as compared to the historical climate. The non-stationarity in climate can be linked directly to precipitation properties (Table S2) that determine whether WPE will yield greater focused recharge. By comparing the projected changes to climate with Fig. 3, it is noted that in systems with high bare soil, the CSIRO model with a high average storm intensity and low annual precipitation results in the most *T*_*L*_. Along the thicketization pathway, the HadGEM2-ES model predicts the most *T*_*L*_ due to its higher annual precipitation which overcomes the canopy interception from the higher amount of shrub cover. Interannual variability in the *T*_*L*_ representing a range of potential climate and WPE impacts demonstrates that WPE largely determines the directionality of changes (Fig. S4). Changes to individual precipitation properties, such as an increase in the average daily storm size or the average annual precipitation, can have a larger impact than WPE on transmission losses (Fig. S5). GCMs do not predict such drastic changes to precipitation properties, however, so when the range of likely changes to precipitation properties is considered WPE is shown to be the primary driver of changes to *T*_*L*_ (Fig. S6).

### Implications of WPE for groundwater sustainability

Woody-plant encroachment into grasslands is often considered a negative outcome and associated with land degradation. Nevertheless, our modeling results illustrate that xerification in a landscape leading to high cover of bare soil can yield increased *T*_*L*_, leading to focused groundwater recharge. At the same time, plant available water is reduced, providing an advantage to shrubs in their competition with grasses^56,57^ and yielding a positive feedback loop that promotes further WPE and focused recharge. In contrast, the thicketization pathway decreases channel losses by increasing hillslope infiltration and evapotranspiration, suggesting that woody plant encroachment in humid regions will not enhance groundwater recharge. Some studies have hypothesized that increases to streamflow in upland systems may result in supplemental water deliveries to downstream ecosystems^16^. Our results suggest that although both WPE and climate change may increase runoff production on hillslopes, this runoff is absorbed in first order channels and does not subsidize downstream ecosystems.

Under a changing climate, the impact of WPE on the dryland water budget is more important than the climate change signal. However, a climate-induced non-stationarity in the hydrologic response emerges for each WPE pathway. For the xerification pathway, water budget components are highly sensitive to changes in extreme precipitation events, whereas the thicketization pathway is most sensitive to changes in total precipitation. We illustrate how this global phenomenon can affect groundwater aquifers through positive (xerification) or negative (thicketization) feedbacks linked to ecohydrological processes. Given that groundwater is the major freshwater resource in many drylands, land managers should consider how woody plant encroachment could affect aquifer sustainability. Because groundwater recharge is potentially more strongly linked to the vegetation state than climate change, WPE should become part of the discourse about management of dryland aquifers in the future.

## Methods

### Ecohydrological Process Modeling

Numerical simulations were performed using the TIN-based Real-time Integrated Basin Simulator, tRIBS^58,59^, a fully-distributed, physically-based ecohydrological model. The tRIBS framework captures high-resolution topography, soil type, vegetation, and meteorological conditions affecting the land-phase of the hydrologic cycle. For each model element, a range of processes that track the watershed response are calculated, including: (1) canopy interception and evaporation; (2) infiltration, soil moisture redistribution, and runoff generation; (3) evaporation from bare soil and transpiration from vegetation; (4) shallow subsurface flow; and (5) overland and channel flow. The model is also capable of ingesting time-variable vegetation parameters^60^ and the modeling domain can be partitioned into subdomains for parallel computing^61^.

We briefly describe the infiltration, runoff generation, channel transmission losses, and vegetation representations for dryland systems with shallow soils. Each model element has a heterogeneous, sloped soil column above a semi-impermeable carbonate layer^45^. A modified version of the Green-Ampt equation that represents unsaturated flow in layered soils is used to calculate infiltration^58,62^. Precipitation pulses lead to single infiltration fronts that interact with antecedent soil water to impact runoff generation and subsequent infiltration. Soil water is redistributed laterally based on topographic gradients. Following storm events, soil water is depleted through bare soil evaporation and plant transpiration to meet the atmospheric demand via closing the energy balance using the Penman-Monteith equation^58^. A vegetative fraction for each model element determines the fractions of bare soil evaporation and transpiration and is treated as a species-dependent parameter that varies with observed plant phenology^46^.

tRIBS simulates runoff generation as either Hortonian (infiltration excess) or Dunnian (saturation excess) processes depending on the wetness state in hillslope soils^58^. Groundwater exfiltration and perched return flow can also be simulated, but are not observed in this system^46^. Runoff is transported to the watershed outlet by first routing the runoff along each hillslope in the direction of steepest descent based on the hillslope path length and a velocity dependent on the downstream channel discharge^58,62^. In the channel network, river routing is simulated using a one-dimensional, finite element, kinematic wave approximation using the Manning’s equation for rectangular cross sections. Recent modifications to the model allow for channel transmission losses of hillslope-derived runoff that account for the impact of capillary forces on infiltration in ephemeral channels during the initial period of infiltration^46^, termed the transient period. A key limitation of this approach is that this model is not a fully integrated surface water-groundwater model. Therefore, transmission losses that are lost from the model domain cannot be accessed by deep-rooted shrubs. Additionally, all shrubs in the modeling framework have their roots limited to the soil layer (top 50 cm) and the carbonate layer is treated as a fully impermeable unit. While these assumptions result in a simplified system, *in situ* observations suggest that they are reasonable approximations and will not significantly alter conclusions drawn from the model^37,63^.

### Woody Plant Encroachment Scenarios

This study was performed in a mixed shrubland of the Jornada Experimental Range USDA-LTER site of southern New Mexico^45^. The study watershed has undergone woody plant encroachment since 1850^43^ leading to the current state consisting of creosote bush (*Larrea tridentata*), honey mesquite (*Prosopis glandulosa* Torr.), several perennial bunchgrass species (*Muhlenbergia porteri*, *Pleuraphis mutica*, and *Sporobolus cryptandrus*), and other shrubs (*Parthenium incanum*, *Flourensia cernua*, and *Gutierrezia sarothrae*). The model was parameterized to ensure accurate representation of shrubs and grasses. When conducting WPE scenarios, however, a generic shrub class was used based upon the time-variable parameters and phenology of mesquite shrubs. No significant differences in the water budget were obtained when a set of mixed shrubs were represented.

To generate the WPE scenarios, we constructed random distributions of shrubs, grasses, and bare soil to meet plant cover specifications (Fig. 1c,d). These were generated within ArcGIS to create spatially distributed raster datasets. Several random realizations of shrub distribution for the same total shrub cover as the current observed state yielded no significant difference in the hydrologic response (Fig. S7). Plant cover specifications were developed to follow the two WPE pathways. Xerification better represents dry conditions at the study site where WPE results in high bare soil cover. To achieve these, we developed relations between aboveground net primary production (ANPP) and plant cover for grasses and shrubs. Total ANPP does not change with WPE^64,65^ in arid environments where xerification occurs, but the relation between biomass and percentage cover is steeper for shrubs than for grasses; thus, to maintain constant ANPP with WPE, there is an increase in bare soil. Thicketization occurs in more humid environments where grasses are replaced by shrubs since there is sufficient precipitation to support increased ANPP (Fig. S8).

### Model Scenario Simulations

We performed the hydrologic simulations for a period that corresponds with hydrologic monitoring at the site^45^: July 1, 2010 – September 30, 2016 (6.25 years), a total of 7 growing seasons (1 July to 1 October). Meteorological forcings consisted of observed values of solar radiation, wind speed, air temperature, relative humidity, and barometric pressure at 30-minute intervals and applied uniformly to the watershed^46^. Precipitation at 30-minute resolution was derived from four rain gauges in the watershed^45^. Simulations were performed with a spatial resolution of 1 m resulting in ~47,000 computational elements requiring 25 CPU hours per year of simulation. Parallel computations were used to decrease simulation time based on subdomain partitioning of the channel network into 8 regions^61^. A base case consisting of observed vegetation at the study site was calibrated and validated using extensive observations of soil moisture, streamflow, evapotranspiration, and the energy balance^46^. Static model parameters are presented in Table S1. Vegetation parameters include time variable phenology based on phenocam measurements from the study site^46^. The model outputs of interest are the water budget components of evapotranspiration, streamflow, and transmission losses. Transmission losses are defined as deep percolation in the channel network and can occur even when insufficient streamflow develops to exit the watershed.

Climate change scenarios rely on stochastic downscaling of general circulation models (GCMs) to produce representative realizations of potential future climates in the region^66–68^. This technique applies delta change values to the statistical properties of historical precipitation and air temperature based on the differences between current and future periods. We obtained projections from the Coupled Model Intercomparison Project version 5 for three GCMs shown to be effective for desert regions^69^: (1) CNRM-CM5, (2) CSIRO Mk3.6.0, and (3) HadGEM2-ES. We selected single realizations from each model that represent a late century (2085–2100) period under the representative concentration pathway (RCP) 8.5. The 15-year period was selected to match the length of a historical period from NLDAS-2^70^ (1990–2005). We used the statistical properties of the 15-year periods to generate hourly forcing data, representative of the three GCMs and the historical conditions, over synthetic 100-year periods. These forcings should be considered representative realizations of the climate under stationary historical and late century conditions. Due to computational limitations, we selected three WPE scenarios to combine with the climate change cases: (1) 70% grass (0% shrub), (2) 0% grass xerification (35% shrub), and (3) 0% grass thicketization (70% shrub). Each vegetation state was run with the stochastically generated forcings representing historical conditions and late 21^st^-century conditions. We note that hourly precipitation values suppress finer-scale variations which are important for the short duration, high-intensity events leading to channel transmission losses at the site. Because of this, simulations using the long-term synthetic data are used only to compare the relative importance of WPE and climate change on the dryland water budget.

## Supplementary information

Supplementary information.
