# Supplementary material for: Woody Plant Encroachment has a Larger Impact than Climate Change on Dryland Water Budgets
Source: Sci Rep. 2020 May 15;10:8112. doi: 10.1038/s41598-020-65094-x (PMC7229153; doi:10.1038/s41598-020-65094-x)
Supplement: Supplementary file 1 — Supplementary information. [file 41598_2020_65094_MOESM1_ESM.docx]

**Supplemental Information for:**

**Woody Plant Encroachment has a Larger Impact than Climate Change on Dryland Water Budgets**

by

A. P. Schreiner-McGraw, E. R. Vivoni, H. Ajami, O. E. Sala, H. L. Throop, and D. P. C. Peters

| Parameter | Variable (unit) | Land surface classification | | |
| --- | --- | --- | --- | --- |
|  |  | Bare soil | Shrub | Grass |
| - | Area (%) | 66 | 29..7 | 4.3 |
| *K_s_* | Saturated hydraulic conductivity (mm/hr) | 2 | 10 | 6 |
| *n* | Soil porosity (dimensionless) | 0.5 | 0.5 | 0.5 |
| *θ_s_* | Saturated soil moisture content (dimensionless) | 0.23 | 0.23 | 0.23 |
| *θ_r_* | Residual soil moisture content (dimensionless) | 0.02 | 0.02 | 0.02 |
| *m* | Pore size distribution index (dimensionless) | 0.6 | 0.6 | 0.6 |
| Ψ*_b_* | Air entry bubbling pressure (mm) | -0.0001 | -0.0001 | -0.0001 |
| *As* | Saturated anisotropy ratio (dimensionless) | 1 | 1 | 1 |
| *Au* | Unsaturated anisotropy ratio (dimensionless) | 50 | 50 | 50 |
| *k_s_* | Soil heat conductivity (J/msK) | 5 | 5 | 5 |
| *C_s_* | Soil heat capacity (J/m^3^K) | 200000 | 200000 | 200000 |
| *Zr* | Soil depth (m) | 0.5 | 0.5 | 0.5 |
| *h* | Vegetation height (m) | 0 | 1.5 | 0.3 |
| *K_sc_* | Channel saturated hydraulic conductivity (mm/hr) | 663 | | |
| *K_tc_* | Channel transient hydraulic conductivity (mm/hr) | 46,410 | | |
| *τ* | Transient time (hours) | 1 | | |

**Table S1**: Static model parameters used within this study. Sources for parameters and details regarding model calibration can be found in ref. 37.

| Property [units] | Historical | HadGEM2-ES | CSIRO Mk3.6.0 | CNRM-CM5 |
| --- | --- | --- | --- | --- |
| Precipitation [mm/yr] | 259.0 | 300.0 | 220.0 | 288.0 |
| Average S_p_ [mm] | 6.0 | 9.2 | 7.1 | 4.7 |
| Storm Frequency [N_e_/yr] | 45.8 | 32.2 | 31.0 | 65.3 |
| Extreme Event Frequency [N_e_/yr] | 1.5 | 3.0 | 2.0 | 1.1 |
| Average Precipitation Intensity [mm/hr] | 2.3 | 2.0 | 3.6 | 1.8 |

**Table S2**: Average precipitation properties for the 100-year stochastic representations of projected climate.


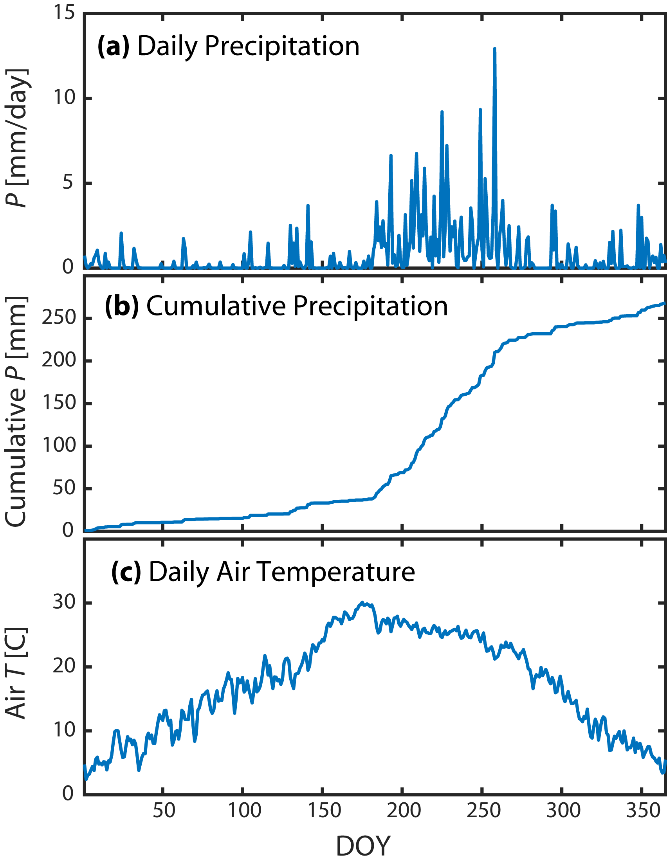


**Figure S1**: The average annual cycle of precipitation and air temperature forcings for the 6.25-year observation period.


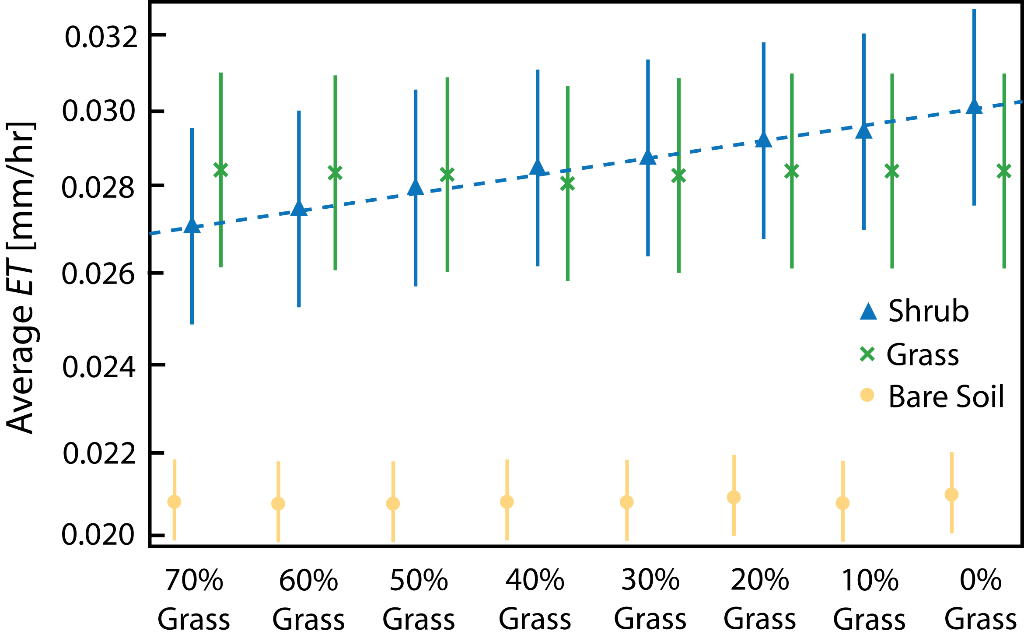


**Figure S2:** The average hourly *ET* from different vegetation classes and bare soil along the xerification pathway. Points represent the mean value and error bars represent the standard deviation among all model elements of that type. The statistically significant (*p* < 0.0001) linear relation between the average *ET* for shrubs and the grass cover percent is shown as a dashed line.


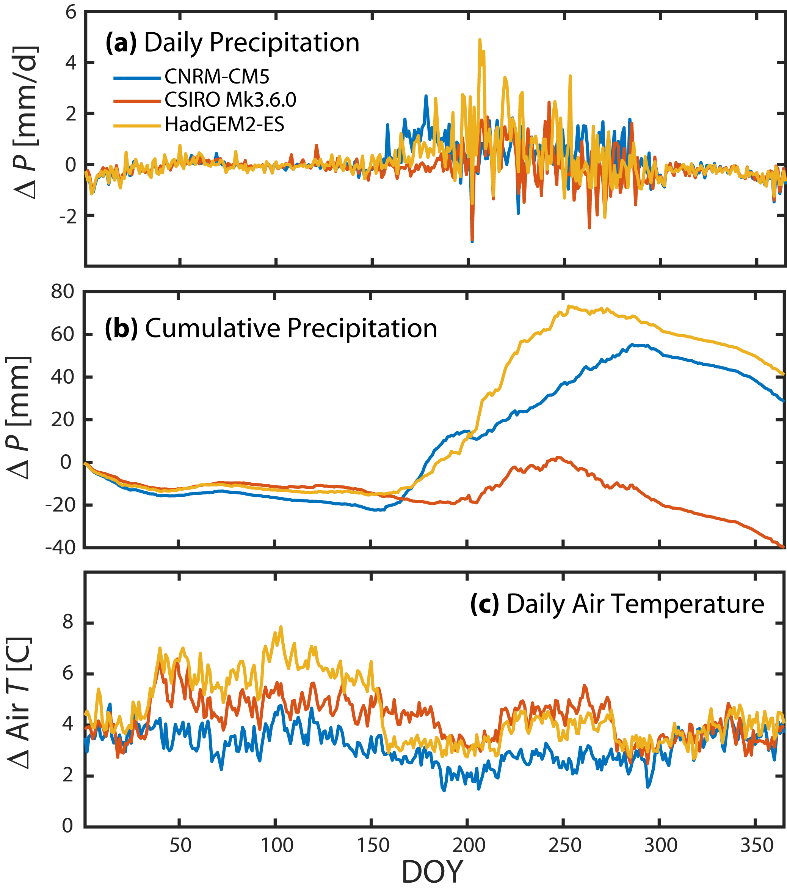


**Figure S3:** Differences in the average annual cycles of precipitation and air temperature between the three GCMs and the historical conditions from NLDAS-2.


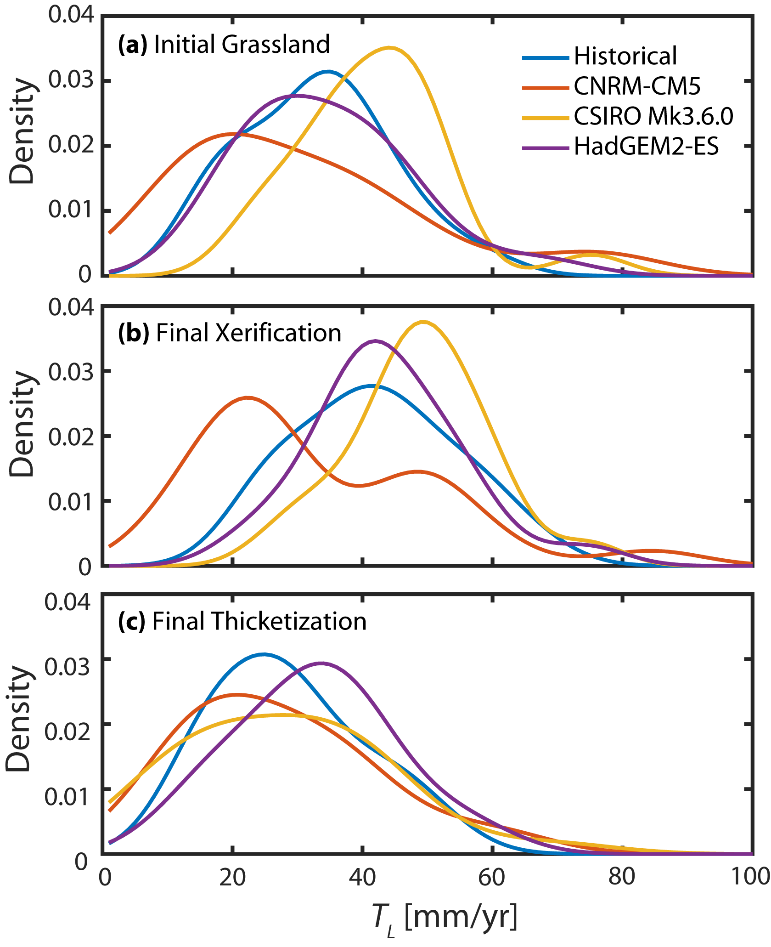


**Figure S4:** Probability density distributions of annual transmission losses (*T_L_*) for each of the combined WPE and climate change scenarios for the (a) initial grassland (70% grass, 0% shrub), (b) final xerification (0% grass, 35% shrub), and (c) final thicketization (0%grass, 70% shrub).


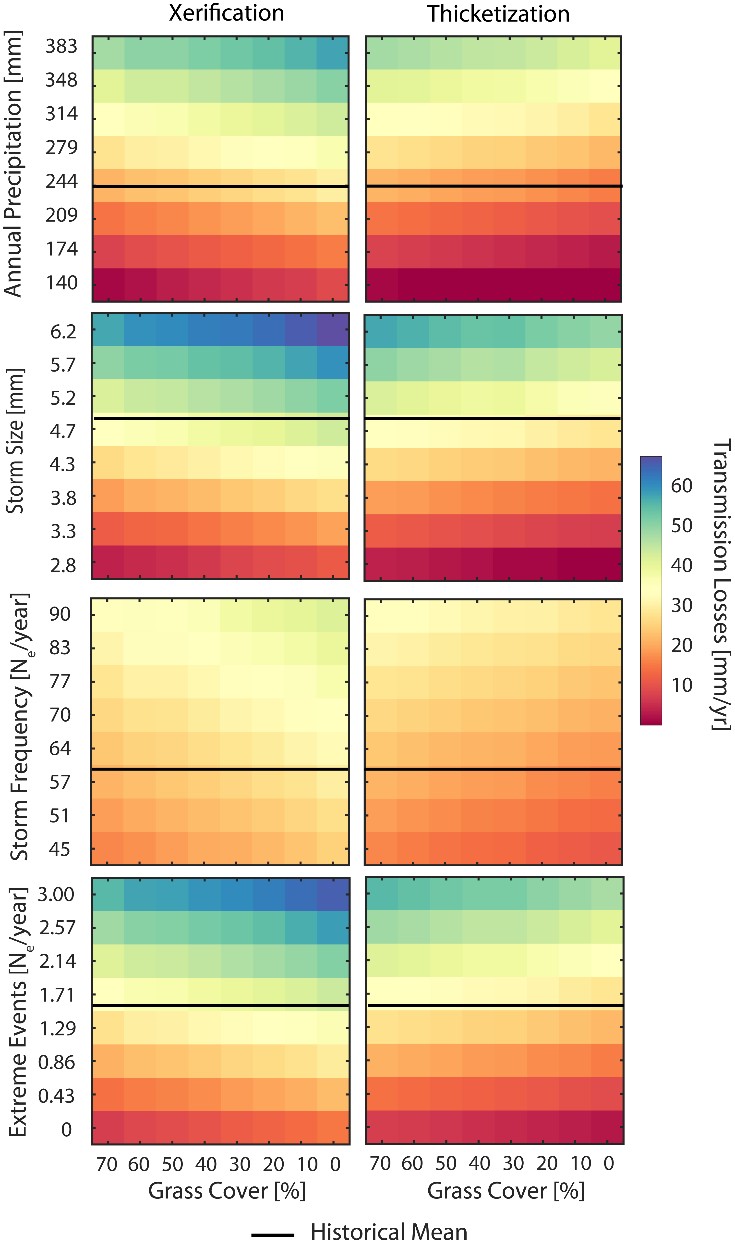


**Figure S5:** Controls of precipitation properties on transmission losses across the range of grass cover scenarios for the xerification (left column) and thicketization (right column) pathways. Outputs from the 6.25-year observed simulations are used to create the relations. Extreme events are defined as daily accumulations of precipitation greater than 25 mm, which corresponds to the 97^th^ percentile of precipitation event.


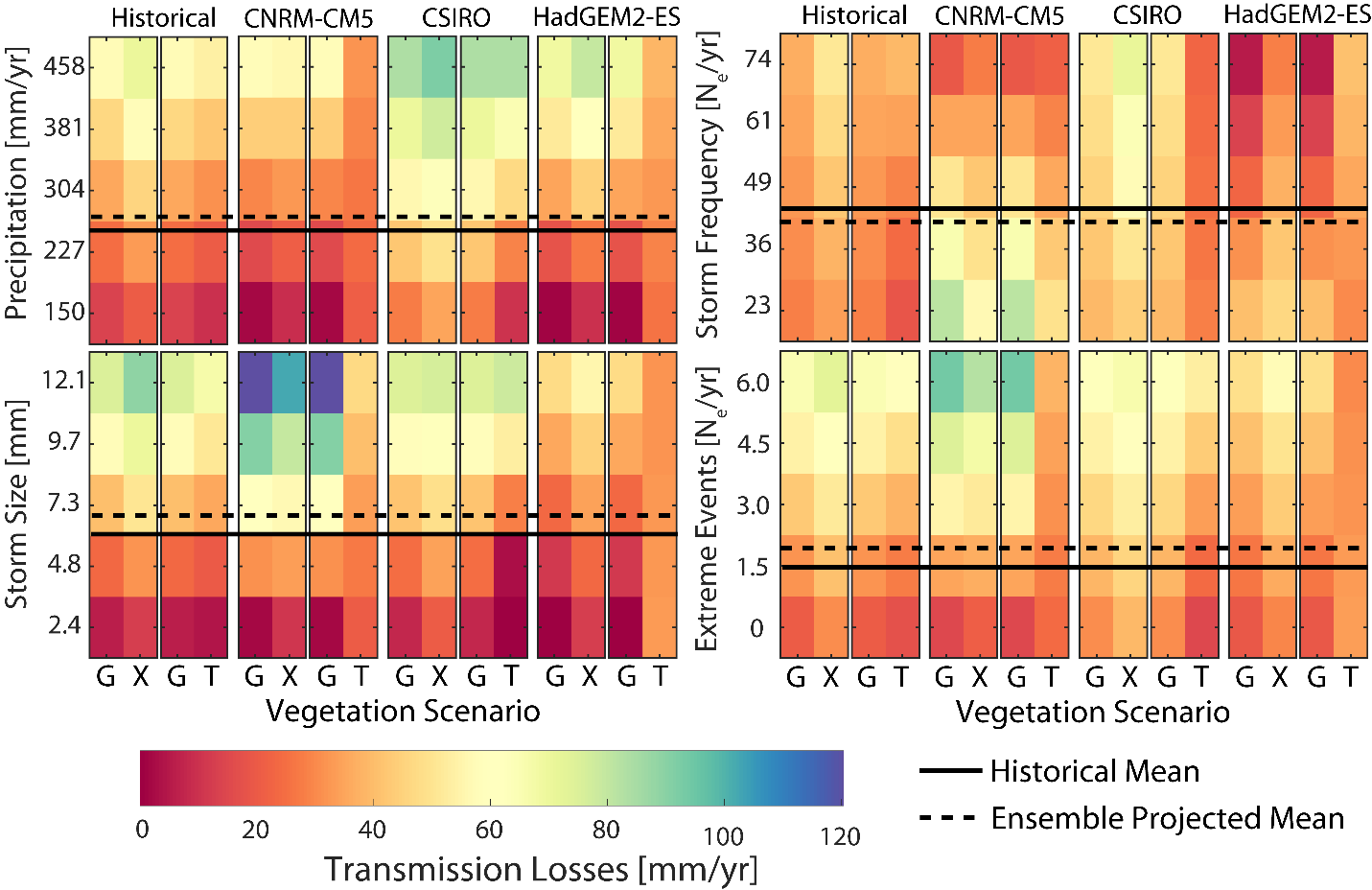


**Figure S6:** Controls of precipitation properties on transmission losses across the range of climate change scenarios for the initial grassland (G; 70% grass, 0% shrub), final xerification (X; 0% grass, 35% shrub), and final thicketization (T; 0% grass, 70% shrub) states. Outputs from the 100 year stochastically downscaled simulations are used to create the relations. The historical mean value for each precipitation property is shown in a solid black line, while the projected ensemble average of the 3 GCMs used is shown in a black dashed line. Extreme events are defined as daily accumulations of precipitation greater than 25 mm, which corresponds to the 97^th^ percentile of precipitation event.


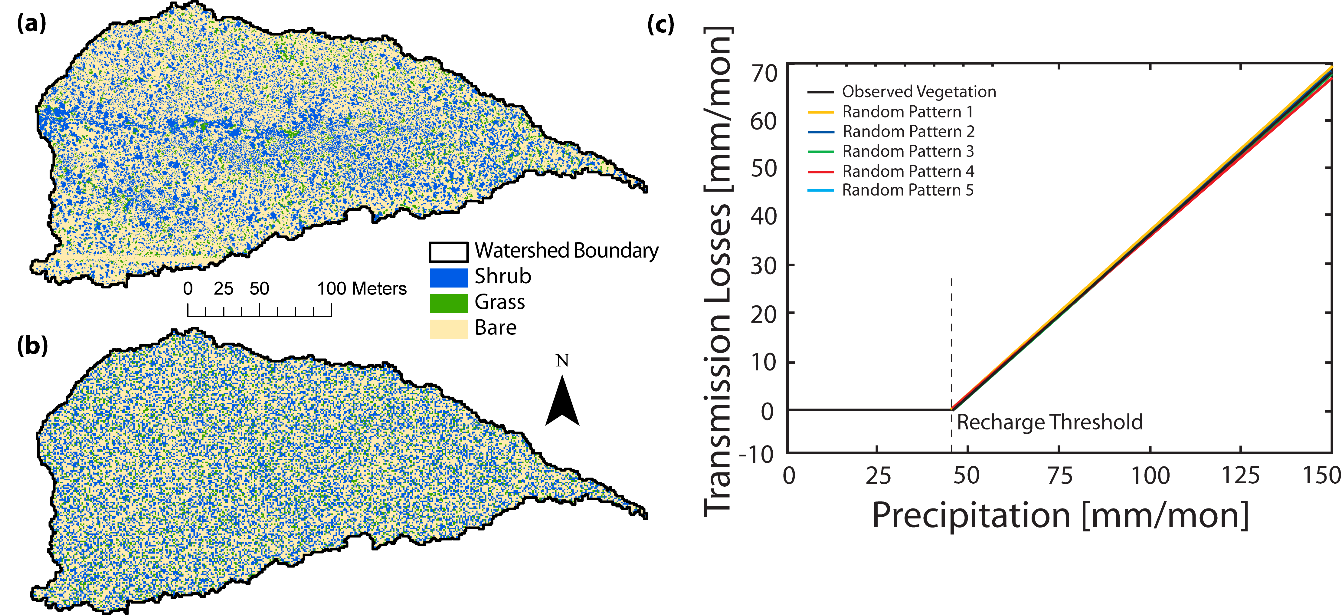


**Figure S7:** (a) The observed vegetation state at the study site. (b) A randomly generated vegetation state that matches the percent coverage of each class in the observed state. (c) Simulated transmission losses for a range of monthly precipitation values using the observed vegetation pattern and 5 randomly generated vegetation patterns with the same plant cover for each class within each realization.


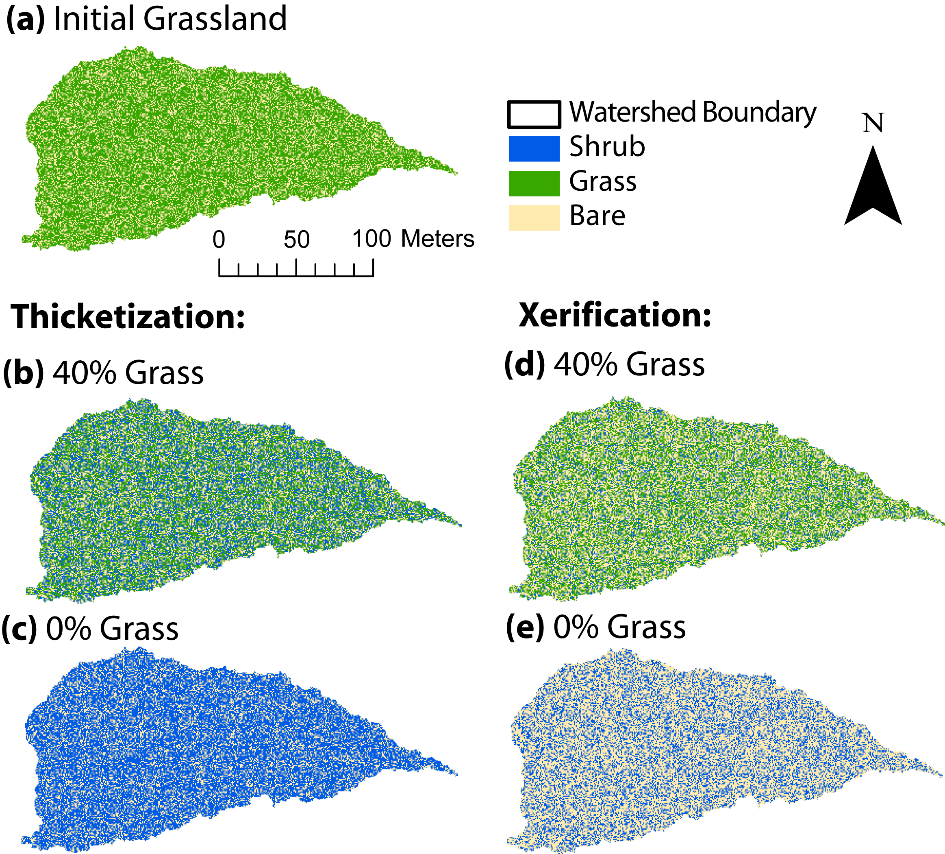


**Figure S8:** Examples illustrating the random distributions of bare soil, grass, and shrub cover for the initial grassland (a), two vegetation states along the thicketization pathway (b,c) and two vegetation states along the xerification pathway (d, e).
